# Should Patients with Renal Cell Carcinoma and Pathological Nodal Invasion Be Classified As Having Stage IV Disease?

**DOI:** 10.1245/s10434-022-12979-y

**Published:** 2023-06-08

**Authors:** Kai-Jie Yu, Sy-Yuan Chen, Po-Hung Lin, Chung-Yi Liu, Aron Y. Joon, Yu-Kuan Yang, I.-Hung Shao, Hung-Chen Kan, Yuan-Cheng Chu, Liang-Kang Huang, Ying-Hsu Chang, Cheng-Keng Chuang, Wen-Hui Weng, See-Tong Pang

**Affiliations:** 1 Division of Urology, Department of Surgery, Linkou Chang Gung Memorial Hospital, Taoyuan, Taiwan; 2 Department of Urology, New Taipei Municipal Tucheng Chang Gung Memorial Hospital, New Taipei City, Taiwan; 3 College of Medicine, Graduate Institute of Clinical Medical Sciences, Chang Gung University, Taoyuan, Taiwan; 4 Department of Chemical Engineering and Biotechnology, Graduate Institute of Biochemical and Biomedical Engineering, National Taipei University of Technology, Taipei, Taiwan; 5 Department of Biostatistics, The University of Texas MD Anderson Cancer Center, Houston, TX USA

## Abstract

**Background:**

Lymph node invasion is associated with poor outcome in patients with renal cell carcinoma (RCC).

**Patients and Methods:**

Patients with RCC within a single center from 2001 to 2018 were retrospectively obtained from the Chang Gung Research Database. Patient gender, physical status, Charlson Comorbidity Index, tumor side, histology, age at diagnosis, and body mass index (BMI) were compared. The overall survival (OS) and cancer-specific survival (CSS) of each group were estimated using the Kaplan–Meier method. Log-rank tests were used to compare between the subgroups.

**Results and Conclusions:**

A total of 335 patients were enrolled, of whom 76 had pT_3_N_0_M_0_, 29 had pT_1–3_N_1_M_0_, 104 had T_1–4_N_0_M_1_, and 126 had T_1–4_N_1_M_1_ disease. Significant OS difference was noted between pT_3_N_0_M_0_ and pT_1–3_N_1_M_0_ groups with 12.08 years [95% confidence interval (CI), 8.33–15.84] versus 2.58 years (95% CI, 1.32–3.85), respectively (*P* < 0.005). No significant difference was observed in OS between pT_1–3_N_1_M_0_ and T_1–4_N_0_M_1_ groups with 2.58 years (95% CI, 1.32–3.85) versus 2.50 years (95% CI, 1.85–3.15, *P* = 0.72). The OS of N_1_M_1_ group was worse than that of N_0_M_1_ group with 1.00 year (95% CI, 0.74–1.26) versus 2.50 years (95% CI, 1.85–3.15, *P* < 0.05). Similar results were also observed in CSS. In summary, we claim that RCC with lymph node (LN) invasion should be reclassified as stage IV disease in terms of survival outcome.

According to the eighth edition of the American Joint Committee on Cancer (AJCC) tumor–node–metastasis (TNM) staging system,^1^ patients with renal cell carcinoma (RCC) with T_1–3_N_1_M_0_ or T_3_N_0_M_0_ status are classified as stage III, while T4 and M1 status are classified as stage IV. A recent study reported that the overall survival (OS) of patients with stage III cancer with pathologic nodal involvement and those without was 2.4 years [95% confidence interval (CI) 1.7–4.1 years] and 10.2 years (95% CI, 8.7 years–not applicable), respectively.^2^ The study also pointed out that the survival rate of patients with node-positive RCC was similar to that of patients with stage IV disease instead of stage III.

Lymph node (LN) invasion is present in about 4.7% of patients with RCC.^3^ As a vital prognostic predictor in patients with RCC, LN invasion affects the survival outcome and disease aggressiveness.^4,5^ According to current guidelines, LN dissection (LND) does not improve oncological outcomes in patients with clinically node-positive disease; however, it may be beneficial for precise staging.^6,7^

Survival impact of LN invasion was noted in other genitourinary tract malignancies, such as bladder cancer, and it was also the pivot for the success of treatment.^8^ Studies that focus on whether LN invasion has impact on survival must be conducted precisely to find out the divergent presentation according to treatment stratification. As the most recognized staging system, TNM classification is consistently revised via including more stratification to reflect the outcomes according to the rapid pace of modern treatment.^9^ As the third most common genitourinary tract malignancy, approximately 403,262 RCC cases were newly diagnosed globally, with 175,098 deaths in 2018,^10^ and incidence exhibiting an increasing trend as well.^11^ With rapid eruption of systemic treatment in the recent decade, continuous monitoring and revision of the staging system is mandatory to provide most updated treatment guidance and risk stratification accordingly.^12^

Currently, there is only one study advocating that LN invasion should be considered as stage IV, and more than 70% of the study population was Caucasian.^2^ The result was not formally verified in the Asian population, and previous studies reported the difference of survival between races with RCC.^13^ Our study provides the result for Asian patients and verifies the detrimental impact of LN invasion on survival outcome in RCC.

## Patients and Methods

### Data Collection

Data for this study were retrospectively collected from the Chang Gung Research Database (CGRD) after obtaining approval from the Institutional Review Board of the Chang Gung Medical Foundation. The CGRD, an encrypted database of electronic medical records from Chang Gung Memorial Hospital, covers approximately 6% and 10% of outpatient and hospitalized patients, respectively, in Taiwan.^14^ The CGRD is more accurate than other nationwide databases in terms of information; for instance, it contains complete pathological and laboratory reports.^15^ In addition, the CGRD provides key information such as diagnostic codes from both outpatient and inpatient departments, pathological reports, records of medical orders, procedures, recorded vital signs, nursing records, and perioperative condition during anesthesia. In this study, we recorded and stored all essential information by using the structured query language database. We excluded duplicated data by following a standard quality control process.

### Methods

**Fig. 1:**
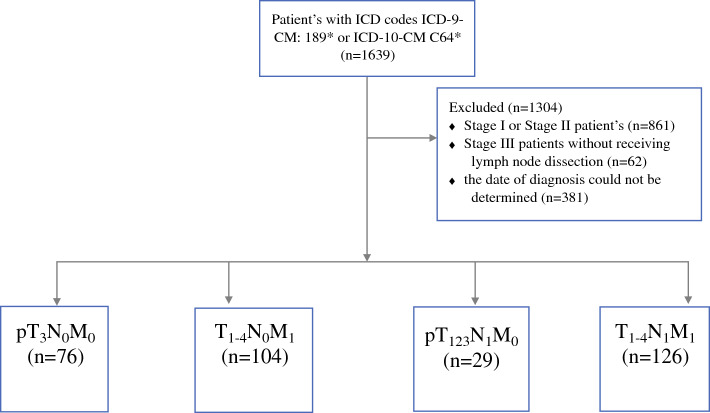
CONSORT diagram of the study

From the CGRD, we retrospectively obtained the data of patients with RCC from one center between 2001 and 2018. The diagnosis of RCC was reviewed and identified according to the International Classification of Disease, Clinical Modification, Ninth Revision (ICD-9-CM) code 189* and the International Classification of Disease, Clinical Modification, Tenth Revision (ICD-10-CM) code C64* assigned by physicians. We only included patients with stage III or IV RCC in this study. The eighth edition of the 2018 AJCC staging system was used for the TNM staging of patients.^1^ We excluded patients with missing information for diagnosis or surgery in the database. The flow of our study design is displayed in our CONSORT diagram (Fig. 1). Our study design was reviewed and approved by the Center for Big Data Analytics and Statistics (Grant CLRPG3D0045) at Chang Gung Memorial Hospital.

In this study, patient characteristics are summarized as numerical and categorical variables, namely the American Society of Anesthesiologists (ASA) physical status classification, Eastern Cooperative Oncology Group Performance Status (ECOG-PS) score, and Charlson Comorbidity Index. In line with other methodological studies, the Charlson Comorbidity Index was calculated using ICD-9-CM and ICD-10-CM codes in both outpatient and inpatient departments.^16,17^

### Statistical Analysis

We used R (version 3.3.0, 2016-05-03) to perform statistical analyses. To compare patient characteristics between subgroups, we used Fisher’s exact test for categorical variables, namely sex, American Society of Anesthesiologists (ASA) physical status classification, ECOG-PS score, Charlson comorbidity index, tumor side, and cell type, and the Mann–Whitney–Wilcoxon test for continuous and ordinal variables, namely age at diagnosis and body mass index (BMI). OS was defined as the interval between date of RCC diagnosis and date of death from any cause or last follow-up date for patients who were still alive. Cancer-specific survival (CSS) was defined as the interval between date of RCC diagnosis and date of death due to RCC. Patients who died of other causes were censored at date of death, whereas patients who were alive were censored at last follow-up date. The OS and CSS of each group were estimated using the Kaplan–Meier method. The Mantel–Cox log-rank test was performed to compare OS and CSS between subgroups. Finally, for OS and CSS, we used the univariate Cox proportional-hazards model for important clinical variables. Subsequently, we included statistically significant variables (*P* < 0.05) in the multivariate Cox proportional-hazards model.

## Results

### Patient Characteristics

Of the 335 patients included in this study, 76 had pT_3_N_0_M_0_, 29 had pT_1–3_N_1_M_0_, and 230 had metastatic disease (104 and 126 had T_1–4_N_0_M_1_ and T_1–4_N_1_M_1_ disease, respectively). The median age of patients was 60 years, and the median Charlson Comorbidity Index was 4. Most patients had an ECOG-PS score of 0 or 1 (87% in total). Table 1 summarizes the characteristics of subgroups included in this study.

**Table 1:** Characteristics of patients with PT_3_N_0_M_0_ and PT_1–3_N_1_M_0,_ M_1_ includes T_1–4_N_0_M_1_ (*N* = 104) and T_1–4_N_1_M_1_ (*N* = 126)

	Total	PT_3_N_0_M_0_ (N_0_M_0_)	PT_1–3_N_1_M_0_ (N_1_M_0_)	M1	*P*-value N_0_M_0_ versus N_1_M_0_	*P*-value N_1_M_0_ versus M_1_
Sample size (*N*)	335	76	29	230		
Age (years) when diagnosed, median (range)	60 (11–91)	58 (16–88)	57 (19–85)	62.5 (11–91)	0.576	0.096
Sex, *N* (%)					1	0.820
Male	229 (68.4%)	50 (65.8 %)	20 (69.0 %)	159 (69.1 %)		
Female	106 (31.6%)	26 (34.2 %)	9 (31.0 %)	71 (30.9 %)		
BMI, median (range)	23.7 (12.4–38.3)	23.7 (16.7–31.3)	23.4 (18.3–38.3)	23.7 (12.4–33.2)	0.537	0.784
ASA, *N* (%)					0.174	0.405
≤ 2	124 (44.9%)	38 (51.4 %)	9 (34.6 %)	77 (43.8%)		
≥ 3	152 (55.1%)	36 (48.6 %)	17 (65.4 %)	99 (56.3%)		
ECOG, *N* (%)					1.000	0.385
≤ 1	292 (94.8%)	71 (93.4 %)	28 (100 %)	193 (93.2%)		
≥ 2	16 (5.2%)	6 (6.6 %)	0 (0 %)	14 (6.8%)		
Charlson Comorbidity Index, median (range)	4 (0–10)	4 (2–10)	4 (2–9)	4 (0–10)	0.070	0.818
Tumor side, *N* (%)					1.000	0.843
Right	146 (43.6%)	30 (39.5 %)	12 (41.4 %)	104 (45.2%)		
Left	189 (56.4%)	46 (60.5 %)	17 (58.6 %)	126 (54.8%)		
Cell type, *N* (%)					0.178	0.002
Clear cell	293 (87.4%)	63 (82.9 %)	20 (69.0 %)	210 (91.3%)		
Non-clear cell	42 (12.5%)	13 (17.1 %)	9 (31.0 %)	20 (8.7%)		

Throughout the parameters we have collected in the study, no statistically significant difference was noted between the PT_1–3_N_1_M_0_ versus M_1_ group. No significant difference was observed between patients with pT_3_N_0_M_0_ and those with pT_1–3_N_1_M_0_. However, a significant difference in the cell type was noted between the pT_1–3_N_1_M_0_ and metastatic (M1) groups (*P* = 0.002); patients in the metastatic group had more clear cell tumors. The distribution of demographics, including median age, sex, and comorbidities, was similar among the subgroups.

### Overall Survival

**Fig. 2:**
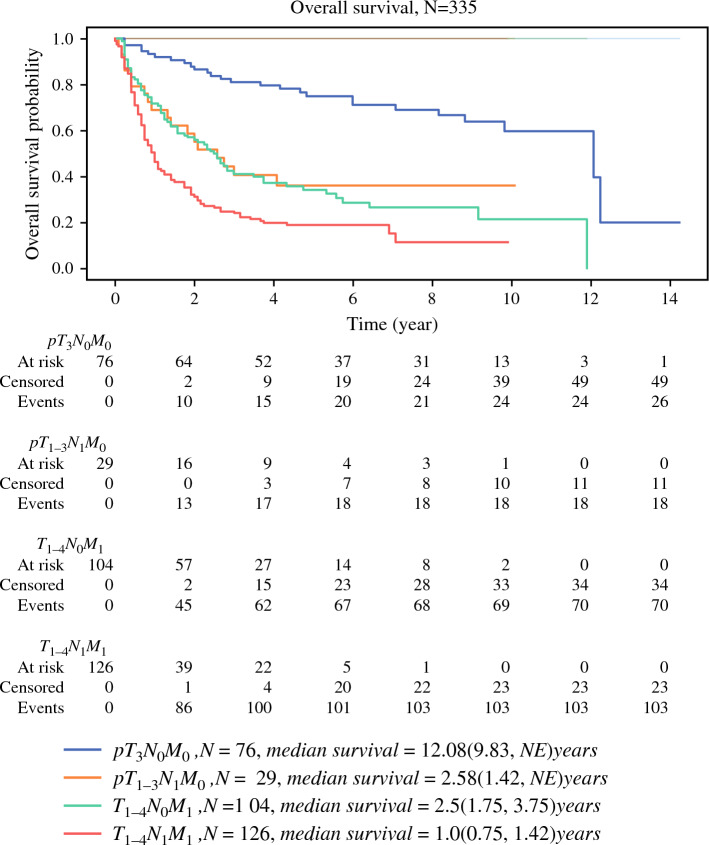
Kaplan–Meier estimates for OS for the patients of the four groups: pT_3_N_0_M_0_, pT_1–3_N_1_M_0_, T_1–4_N_0_M_1_, and T_1–4_N_1_M_1_ disease

Figure 2 shows the Kaplan–Meier estimates of OS in the four subgroups, and Table 2 presents the results of the comparison of median OS and CSS among the four subgroups. The median OS was 2.41 years (95% CI, 1.90–2.93 years). In total, 217 deaths occurred during follow-up. A significant difference was observed in OS among the four subgroups (*P* < 0.005).

**Table 2:** Pairwise comparison: median survival time of OS and CSS (P-values) for the four studied groups using the log-rank (Mantel–Cox) test

OS			CSS		
Groups	Groups	*P*-value	Groups	Groups	*P*-value
pT_3_N_0_M_0_	pT_1–3_N_1_M_0_	< 0.001	pT_3_N_0_M_0_	pT_1–3_N_1_M_0_	< 0.001
pT_3_N_0_M_0_	T_1–4_N_0_M_1_	< 0.001	pT_3_N_0_M_0_	T_1–4_N_0_M_1_	< 0.001
pT_3_N_0_M_0_	T_1–4_N_1_M_1_	< 0.001	pT_3_N_0_M_0_	T_1–4_N_1_M_1_	< 0.01
p T_1–3_N_1_M_0_	T_1–4_N_0_M_1_	0.72	p T_1–3_N_1_M_0_	T_1–4_N_0_M_1_	0.52
p T_1–3_N_1_M_0_	T_1–4_N_1_M_1_	0.02	p T_1–3_N_1_M_0_	T_1–4_N_1_M_1_	0.01
T_1–4_N_0_M_1_	T_1–4_N_1_M_1_	< 0.001	T_1–4_N_0_M_1_	T_1–4_N_1_M_1_	< 0.001
Between all 4 groups		< 0.001	Between all 4 groups		< 0.001

Among patients with stage III disease, the pT_1–3_N_1_M_0_ group had a median OS of 2.58 years (95% CI, 1.42 years to not estimable), which was shorter than that of patients without nodal involvement (pT_3_N_0_M_0_ group, median OS, 12.08 years, 95% CI, 9.83 years to not estimable, *P* < 0.005). However, the difference in OS between the pT_1–3_N_1_M_0_ and T_1–4_N_0_M_1_ groups was nonsignificant (*P* = 0.72), with OS of 2.58 years (95% CI, 1.42 years to not estimable) and 2.50 years (95% CI, 1.75–3.75 years), respectively. Thus, the OS of the pT_1–3_N_1_M_0_ group was similar to that of the metastatic group rather than the pT_3_N_0_M_0_ group.

Even within the metastatic group, patients with LN invasion (T_1–4_N_1_M_1_) exhibited poorer survival outcomes than did those without (T_1–4_N_0_M_1_), with a median OS of 1.00 year (95% CI, 0.75–1.42 years) versus 2.50 years (95% CI, 1.75–3.75 years, *P* < 0.05). These findings indicate that pathologic LN invasion resulted in poorer clinical outcomes in the metastatic groups.

### Cancer-Specific Survival

**Fig. 3:**
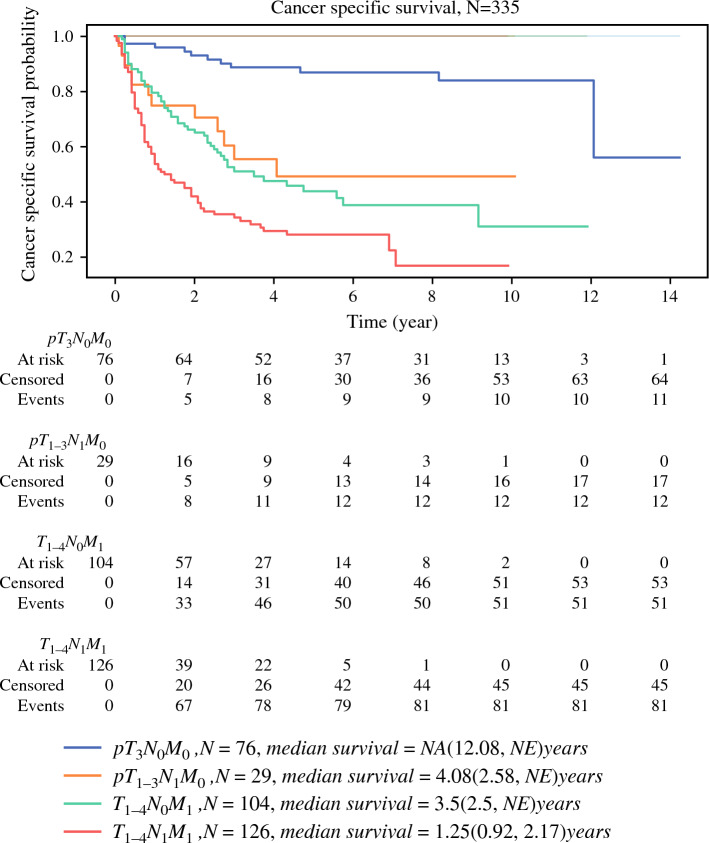
Kaplan–Meier estimates for CSS for the patients of the four groups: pT_3_N_0_M_0_, pT_1–3_N_1_M_0_, T_1–4_N_0_M_1_, and T_1–4_N_1_M_1_ disease

As shown in Fig. 3, the CSS results were similar to those for OS. The median CSS was 4.67 (95% CI, 1.67–7.67) years in the whole cohort. However, the median CSS and its 95% CI could not be calculated for the pT_3_N_0_M_0_ and pT_1–3_N_1_M_0_ groups, as they did not reach median survival. Similar to the results for OS, the difference in CSS between the pT_1–3_N_1_M_0_ and T_1–4_N_0_M_1_ groups was nonsignificant (*P* = 0.52), with a CSS of 4.08 years (95% CI, 2.58 years to not estimable) versus 3.5 years (95% CI, 2.5 years to not estimable); by contrast, the CSS of the pT_1–3_N_1_M_0_ group was significantly shorter than that of the pT_3_N_0_M_0_ group (*P* < 0.05), with a CSS of 4.08 years (95% CI, 2.58 years to not estimable) versus not estimable years (95% CI, 12.08 years to not estimable). The pairwise comparisons of each group are presented in Table 2. Similar patterns of discrimination in CSS were observed between the pT_3_N_0_M_0_ and T_1–4_N_0_M_1_ groups and between the two metastatic groups.

### Univariate and Multivariate Cox Proportional-Hazards Models

Tables 3 and 4 present the fit univariate and multivariate Cox models for OS and CSS in our patients, respectively. This is done by only a subset of patients (*N* = 105, instead of 335) due to the limitation of the database. We found that the ASA physical status, Charlson Comorbidity Index, and pathological nodal disease (pN_1_) were significantly associated with poor OS. Moreover, only the Charlson Comorbidity Index and pN_1_ were predictive of poor CSS.

**Table 3:** Cox proportional-hazards model: overall survival

	Univariate		Multivariate	
	Hazard ratio (95% CI)	*P*-value	Hazard ratio (95% CI)	*P*-value
Age at diagnosis	1.018 (0.998, 1.039)	0.076	Not estimable	Not estimable
BMI	0.978 (0.875, 1.093)	0.695	Not estimable	Not estimable
Charlson Comorbidity Index	1.294 (1.119, 1.496)	< 0.001	1.221 (1.048, 1.423)	0.011
Sex (Reference: M)	0.769 (0.402, 1.474)	0.429	Not estimable	Not estimable
ASA (Reference: ≥ 3)	0.432 (0.224, 0.833)	0.012	0.512 (0.263, 0.997)	0.049
ECOG (Reference: ≤ 1)	0.489 (0.058, 4.112)	0.51	Not estimable	Not estimable
Tumor side (Reference: right)	1.029 (0.552, 1.918)	0.929	Not estimable	Not estimable
Lymph node invasion (Reference: 0)	3.269 (1.751, 6.102)	< 0.001	2.23 (1.14, 4.362)	0.019

Only variables with *P* < 0.05 in univariate models were included in multivariate model

**Table 4:** Cox proportional-hazards model: cancer-specific survival

	Univariate		Multivariate	
	Hazard ratio (95% CI)	*P*-value	Hazard ratio (95% CI)	*P*-value
Age at diagnosis	1.014 (0.986, 1.042)	0.332	Not estimable	Not estimable
BMI	0.982 (0.847, 1.138)	0.805	Not estimable	Not estimable
Charlson Comorbidity Index	1.379 (1.129, 1.683)	0.002	1.302 (1.061, 1.597)	0.011
Sex (Reference: M)	1.236 (0.535, 2.858)	0.62	Not estimable	Not estimable
ASA (Reference: ≥ 3)	0.612 (0.253, 1.481)	0.276	Not estimable	Not estimable
ECOG (Reference: ≤ 1)	0 (0, Inf)	0.998	Not estimable	Not estimable
Tumor side (Reference: right)	1.115 (0.468, 2.659)	0.806	Not estimable	Not estimable
Lymph node invasion (Reference: 0)	4.77 (2.035, 11.178)	< 0.001	3.804 (1.605, 9.019)	0.002

Only variables with *P* < 0.05 in univariate models were included in multivariate model

## Discussion

In clinical practice, an accurate staging system such as TMN staging can help in decision-making for treatment, assessing prognosis and treatment outcomes, and stratifying patients in different treatment modality cohorts.^18^ An updated and well-validated staging system should be established for disease stratification in terms of recurrence or progression risk and for individualized adjuvant therapy plans. The results in the present study revealed that patients with stage III RCC with pathological LN invasion demonstrated poorer OS and CSS than did those without nodal disease. In addition, we found that the survival outcome of patients with stage III RCC with pathological LN invasion was similar to that of patients with stage IV RCC. There are still some points in argument under current practice. First, the number of LNs to be removed was determined according to the template chosen by surgeons for different clinical scenarios, and the number of positive LNs removed depended on the pathologist’s diligence. In addition, LND was not routinely performed with nephrectomy in low-risk patients, and the recent meta-analysis by Wei et al. even demonstrated that unnecessary LND could be detrimental and shorten the CSS if all nonmetastatic patients with RCC underwent LND during radical nephrectomy (HR 1.22, 95% CI 1.05–1.43).^19^ On the contrary, although cytoreductive nephrectomy was no longer the gold standard operation for mRCC in the era of targeted therapy, according to the results from the CARMENA study, it is worth paying attention to the observation that lymph node yield via meticulous dissection could prolong OS in the setting of cytoreductive nephrectomy in patients with mRCC (HR 0.97; 95% CI 0.95–0.99).^20,21^ Thus, surgeons’ selection bias can substantially undermine the clinical application of LND. Accordingly, LN staging can be reliable only if every part of the process is standardized.

Given the advanced nature of nodal disease in RCC, many centers identify patients with a high risk of LN invasion either perioperatively or preoperatively to optimize the efficacy of retroperitoneal LND during nephrectomy. A study proposed that, on the basis of age, symptom classification, and tumor size, the predictive accuracy for pathologic nodal disease was 78.4%.^22^ Another study standardized a pre-surgery model involving a scoring system with four independent predictors, namely tumor stage (cT_3–4_ versus cT_1–2_, score 1 versus 0), clinical nodal status (cN_1_ versus cN_0_, score 1 versus 0), metastasis at diagnosis (cM_1_ versus cM_0_, score 1 versus 0), and clinical tumor size (2–20 cm, score 60–220); the predictive accuracy of the model was reported to be 86.9%.^4^ Another study defined the predictors of LN involvement as follows: intraoperative pathologic feature with nuclear grade 3 or 4, presence of a sarcomatoid component, tumor size ≥ 10 cm, tumor stage of pT_3_ or pT_4_, and histological tumor necrosis.^23^

One important study compared OS and CSS between patients with node-positive stage III and stage IV disease.^2^ The median OS of patients with T_1–3_N_1_M_0_ and patients with stage IV cancer in the aforementioned study was 2.4 years (95% CI, 1.7–4.1 years, respectively). Their results are similar to those of our study, in which the OS of the pT_3_N_1_M_0_ group was 2.58 years (95% CI, 1.42 years to not estimable). These similar findings may indicate that the OS of patients with RCC and node-positive stage III cancer approximates that of patients with stage IV cancer.

Concordantly, our results indicated that, within the metastatic group, the T_1–4_N_1_M_1_ group had poorer OS and CSS than did the T_1–4_N_0_M_1_ group. This finding has not been reported in previous studies and indicates that LN invasion exerted a detrimental effect on survival outcomes even in patients who already had distant metastasis. Although the advantage of LND on survival outcome is still under investigation, and most retrospective reviews did not support the necessity in combination with all nephrectomies, the survival outcome and aggressiveness of disease progression were apparently associated with nodal metastasis.^24–26^ The association of tumor characteristics and lymph node invasion also revealed that the percentage of pathologic nodal disease was higher in advanced tumor stage, with 1.1% in stage T1, 4.5% in T2, and 12.3% in T3.^27^ Interestingly, another study by Sun et al. concluded from the multivariate analysis with the SEER database enrolling 11,374 patients with nonmetastatic RCC who underwent LND that the mortality rate declined from pT1 to pT4 patients with nodal metastases as 6.0-, 3.6-, 3.2-, and 2.0-fold after nephrectomy, respectively (all *P* < 0.001).^28^ These findings verified the lethality of lymph node invasion and it deserved clinical attention as much as other predictors leading to poor outcomes. It is also confirmed in the multivariate Cox model that the Charlson Comorbidity Index and pathological LN invasion are associated with OS and CSS. On the basis of this finding, LN invasion should be considered an independent marker of poor survival outcomes.

Although clinical guidelines indicate LND application in patients with RCC with clinically node-positive disease, the extent of LND and its standard protocol remain controversial due to the complexity of the disease and lack of consensus on dissection. Several studies have demonstrated that LND provides a long-term survival benefit when LN invasion is clinically evident.^29^ In contrast, the results of a randomized phase III trial published in 2009 by the European Organization for Research and Treatment of Cancer Genitourinary Group declared no survival benefit in 30,881 patients who underwent LND in conjunction with radical nephrectomy.^30^ However, criticism regarding the small number of high-risk patients included in the trial undermined its clinical contribution.^31^

Even if a meta‐analysis in 2018 revealed that the relevant literature did not report a survival benefit of LND for M_0_ or M_1_ RCC, evidence on this topic for patients with high‐risk M_0_ RCC has still been inconclusive.^19^ In addition, another review showed no or uncertain therapeutic benefit regarding LND in different groups of patients.^32^ Moreover, recent studies have revealed that LND in RCC did not increase the incidence of surgical complications or major perioperative complications, with only a slight increase in Clavien grade 2 complications.^12,25,33^ Considering the poor OS and CSS of patients with nodal disease, routine LND may still be valuable to make precise staging of patients with clinically high-risk RCC. Additional prospective studies on LND for patients with cT_1–3_N_1_M_0_ are in great need of outcome surveillance to further validate this hypothesis.

Our study has several limitations based on its retrospective nature. First, although patients in our study were treated in one single center, controversy regarding the template indicated that each surgeon’s LND policy might be different. Although most surgeons in our center tended to perform LND in patients with clinical nodal disease according to most experts’ opinion, not all patients in our study underwent adequate dissection. Second, the current study included data from only one tertiary center, and it would be more influential if we could include the whole patient data from all our local branches. It is not open for access currently, but we will initiate further analysis for external validation once the limitation is dismissed. Now that in this present study we analyzed a smaller cohort, it was relatively more unified in terms of the principle of LND than all branch hospitals included.

Regarding the diversity of histology, pathologic grade, age at diagnosis, and underlying diseases in patients with RCC, LN invasion should be taken as an indicator and more attention should be paid in the same way we did for distant metastasis. The detrimental effect could be verified not only for patients with stage III disease, but also for those with distant metastatic disease. Prognostic systems such as TNM staging must be continually revised on the basis of currently available evidence.^34,35^ Our study supported the hypothesis that LN invasion should be reclassified as stage IV disease in RCC.

LN invasion in patients with renal cell carcinoma indicates poor clinical outcome. Stage III patients with pathologic nodal disease should be reclassified as stage IV metastatic disease according to this clinical marker verified with poor outcome.
